# Ecstatic and gelastic seizures related to the hypothalamus

**DOI:** 10.1016/j.ebr.2020.100400

**Published:** 2020-11-05

**Authors:** Kenney Roy Roodakker, Bisrat Ezra, Helena Gauffin, Francesco Latini, Maria Zetterling, Shala Berntsson, Anne-Marie Landtblom

**Affiliations:** a Department of Neuroscience, Neurology, Uppsala University, University Hospital, Uppsala, Sweden; b Department of Neurology and Department of Biomedical and Clinical Sciences, Faculty of Medicine and Health Sciences, Linköping University, Linköping, Sweden; c Department of Neuroscience, Neurosurgery, Uppsala University, Uppsala, Sweden; d Neurology Division, Clinic of Medical Specialist, Motala General Hospital, Motala, Sweden

**Keywords:** Ecstatic seizures, Gelastic seizures, Epilepsy, Hamartoma, Hypothalamus

## Abstract

Ecstatic seizures constitute a rare form of epilepsy, and the semiology is diverse. Previously, brain areas including the temporal lobe and the insula have been identified to be involved in clinical expression. The aim of this report is to review changes in ecstatic seizures in a patient before and after operation for a hypothalamic hamartoma, and to scrutinize the relation to gelastic seizures. In this case, the ecstatic seizures disappeared after surgery of the hamartoma but reappeared eleven years later.

Clinical information was retrospectively obtained from medical records, interviews, and a questionnaire covering seizure semiology that pertained to ecstatic and gelastic seizures. Our findings imply a possible connection between gelastic and ecstatic seizures, originating from a hypothalamic hamartoma. To our knowledge, this location has not previously been described in ecstatic seizures. Gelastic seizures may in this case be associated with ecstatic seizures.

We speculate that patients with ecstatic seizures may have an ictal activation of neuronal networks that involve the insula.

Our case may add information to the knowledge concerning ecstatic seizures.

## 1 Introduction

Ecstatic seizures are considered a rare phenomenon and occur in people with epilepsy experiencing an altered state of consciousness and “feeling of total bliss”. Clinical examples described in the contemporary scientific literature are few. In addition, there are cases from history and religion, such as St Paul and Joan of Arc [1], [2], [3], [4], [5]. A recent review contains all published clinical cases so far, altogether totalling 52 patients [6].

This type of seizures was initially suspected to involve the limbic system evoking “heightened self-awareness”. The patients may describe “absence of time, absence of planning or fear and a positive emotional reinforcement” [6]. The condition may be disregarded in the clinic since some patients can be unwilling to reveal such overwhelming personal feelings to their physicians. Such patients are sometimes reluctant to take medication due to their desire to experience pleasant sensations [7].

Attempts to identify the anatomical structures involved in the complex semiology of ecstatic seizures have brought conflicting results [6], [7]. Previously, the origin of ecstatic seizures has been linked to the medial and lateral *temporal lobe* [7], [8]. There are descriptions of subjects with ecstatic seizures with temporal EEG findings as well as patients with brain tumors located in the anterior temporal lobe [8], [9], [10], [11].

There is now substantial evidence for the involvement of the *insula* in ecstatic seizures [6], [12] provided by brain imaging utilizing multimodal techniques [6], [13], but also supported by cases with direct electrical stimulation of the brain using intracerebral electrodes [14], [15]. The known functions of the anterior–dorsal insula support such a hypothesis [12]. However, there are other patients with ecstatic seizures that have an epileptic focus distant to the insula such as in the left frontal gyrus [16], the right occipital lobe [17] and the parietal lobe [18]. Interestingly, in addition a few patients with generalized epilepsy have experienced ecstatic seizures to confound localization [19].

Some studies have suggested a lateralization in patients with seizures manifesting emotions, especially when the seizure onset zone is in the temporal lobe and the amygdala [20]. Such a lateralization with respect to the insula was reported – with euphoric feelings lateralizing to the left (e.g. mirth), and dysphoric feelings lateralizing to the right (e.g. fear, anger, sadness) [21]. However, discrete lateralization of emotions was unable to be confirmed in a larger study of patients with ecstatic seizures [7].

Gelastic seizures occur in early childhood [22], and are known to be related to hypothalamic hamartomas [22]. The gelastic seizure is described as a “desire or pressure to laugh” that often occurs without any “funny” emotional feeling but may also be associated with a sensation of joy [23]. Seizure onset typically occurs with laughter. The patient is often aware of it, and it may occur daily. The laughter may be the only ictal manifestation during childhood, but is later often followed by multiple focal and generalized seizures due to evolution of epileptogenesis [22], [24]. According to one author, it is difficult to differentiate gelastic seizures due to a hypothalamic hamartoma versus seizures with laughter of frontal or temporal cortical origin [24].

Epileptic activity within pre-existing neuronal wiring of functional networks causes seizures [25], [26]. Consequently, ecstatic seizures rely on abnormal activation, probably in a dynamic interplay between the region of onset and connected target areas [12], [27], [28]. This is in agreement with connectomic theories on brain organization and functions, suggesting complex functional connectivity behind each symptom [29], [30], [31], [32].

We sought to investigate changes in ecstatic seizures in one patient before and after the operation of a hypothalamic hamartoma. We aimed to better understand the origin and propagation of such seizures. A secondary goal was to analyze the relationship between gelastic and ecstatic seizures.

## 2 Case report

### 2.1 Ecstatic seizures in a patient with a hypothalamic hamartoma

Semiology changes related to ecstatic seizures were evaluated over an eleven-year follow-up period including assessment before and after the resection of a hypothalamic hamartoma.

#### 2.1.1 Medical history

A Swedish man born in 1964 to a family with no history of epilepsy or other neurological disorders exhibited paroxysmal episodes of smiling and giggling beginning at the age of one, retrospectively interpreted to represent gelastic seizures. At six years of age, the patient had an accident, which might have been caused by a fit. At the age of eight, he was diagnosed with epilepsy following the onset of focal motor seizures affecting the right half of the body. Soon after, other focal seizures were described with contraction of the corner of the right side of the mouth. At times there were associated features including eye deviation, tachycardia, impaired consciousness, and dysphasia. During adolescence, the patient experienced his first generalized tonic–clonic seizure, and during this time, gelastic seizures became more frequent. The patient’s earliest memories of ecstatic seizures are from the same period.

#### 2.1.2 Clinical investigations, surgical and pharmacological treatment

Pneumoencephalography in childhood revealed atrophy centrally and around the left ventricle. MRI later showed that the left temporal lobe was slightly smaller than the right. In adulthood, he underwent an epilepsy surgery investigation, where MRI revealed a hypothalamic hamartoma in the third ventricle.

Repeated interictal EEGs demonstrated epileptiform activity bilaterally, but most prominent in the left frontotemporal region. Ictal EEG was performed, but no focal ictal onset was identified (See Table 1). Ictal SPECT showed hyperperfusion in the frontal region of the left hemisphere. At the age of 41, the patient had surgery for a left-side predominant hypothalamic hamartoma. The surgical approach was sub-frontal, using lamina terminalis as a surgical corridor. No white matter bundles crossing the region were identified to be involved in the tumor. The surgical resection was not radical and residual hamartoma remained in the corpus mamillare.

**Table 1:** Investigations in a patient with a hypothalamic hamartoma and ecstatic seizures.

Ictal EEG preoperative	Six seizures were discovered using video-EEG during sleep. The head turned slightly to the right followed by clonic jerking of the right arm and leg for 10–15 s. No seizure activity was identified on the EEG because of myogenic artifacts. No post-ictal features were present on EEG after the seizure. During the second ictal registration five seizures were recorded during sleep. During these seizures, the patient would raise his right arm and flex his left arm. Tonic contraction of the right side of the face was followed by evolution to a convulsive seizure. Normal interictal EEG was followed by muscle artifacts without a focal or lateralized ictal onset identifiable. At the end of the seizure there was generalized rhythmic activity that was more pronounced over the left hemisphere.

Ictal SPECT preoperative	Ictal single photon emission computed tomography (SPECT) injection was performed seconds after the start of a seizure. The semiology reflected a focal tonic seizure involving the right side of the face as if the patient was laughing. He was aphasic and the pupils were dilated bilaterally. Regional cerebral hyperperfusion was present in the cortex just above the level of the basal ganglia in the left frontal lobe.

Ictal EEG postoperative	During sleep thirteen seizures with stretching of the right arm, lasting approximatively five seconds, were identified. Only muscle artifacts were seen on EEG. During a few tonic seizures, lasting only a few seconds, EEG showed rhytmic generalized activity.

Ictal SPECT postoperative	Regional cerebral hyperperfusion during ictal SPECT localized to the left frontal lobe in front of the central sulcus. The semiology consisted of elevation of the right arm and rotation of his head to the right followed by focal motor tonic-clonic seizures for up to 3 min.

Postoperatively, a video-EEG monitoring was performed, though no ictal onset was visible due to obscuration by muscle artifacts during the seizures. The ictal SPECT was repeated and confirmed earlier findings showing focal hyperperfusion in left frontal lobe (See Table 1).

Since childhood, the patient was treated with antiseizure medication in polytherapy. Different combinations involving carbamazepine, valproate, phenytoin, oxcarbazepine, clonazepam, topiramate, vigabatrin and zonisamide were utilized. A vagus nerve stimulator (VNS) was implanted in 2012, with improved seizure control. In 2015, antiseizure medications included levetiracetam, primidone and lacosamide.

#### 2.1.3 Questionnaire, medical records and radiological data

We developed a questionnaire on the subjective experiences of epileptic seizure symptoms, aimed for a larger upcoming study on epileptic seizure semiology. However, it is still unvalidated and unpublished. Three interviews with the patient were conducted over time to obtain clinical information on evolving seizure semiology. In addition, serial brain MRIs in 2005 (preoperative), 2006 (postoperative) and 2017 (most recent investigation) were used to reconstruct the tumor size.

## 3 Results

### 3.1 Questionnaire, medical records and patient interviews

The clinical data obtained from the questionnaire, records and interviews together confirmed ecstatic seizures in our patient. In the questionnaire, the patient acknowledged all three criterial symptoms of ecstatic seizures [6]: intense serenity and bliss (“strong feelings of euphoria; happiness without a cause”), enhanced physical well-being (“feelings in the head”) and clarity (“a feeling of hearing and understanding everything”), see citation below.

*“Feelings in the head, a happy feeling without a cause, a pleasant feeling, a dream about something wonderful, a feeling of hearing and understanding everything, like when a dark sky is being lit up by the sun. Words cannot describe it. No other life experience is comparable”* The experience of an ecstatic seizure described by the patient in our study (Patient interview, August 2016).

The *ecstatic seizures* were sometimes preceded by gelastic seizures but had otherwise no particular triggers. According to the patient, the ecstatic seizures may occur in clusters of up to 30 seizures per day during a few days, followed by a few seizure-free weeks or months. They started as a pleasant feeling that would intensify and eventually reach a climax of euphoria before completely fading away. No impairment of conscience was reported. Each seizure lasted for a few minutes. Their intensity often increased during the day to reach a climax in the afternoon and become moderate in the evening.

The *gelastic seizures* occurred in periods 5–10 times per day according to the patient. The frequency was higher during days with ecstatic seizures. No audible laughter or impairment of consciousness was reported by witnesses. The seizures seemed interlinked with the emotional state of the patient in that they could be triggered by emotions as well as reinforce whatever emotion the patient would experience at seizure onset. Melancholy would deepen and happiness would become stronger, although never as intense as during the ecstatic seizures. Seizure symptoms would increase in intensity until reaching a plateau, before fading. The gelastic seizures often fluctuated in intensity over longer time-periods before vanishing completely.

In both ecstatic and gelastic seizures there was sometimes speech impairment and contraction of the corner of the right side of the mouth.

A couple of years before surgery the seizures intensified with frequent focal motor seizures, affecting the right side of the body and face, including problems speaking. There were also nightly tonic-clonic seizures, sometimes several times per week. They were later complicated by a persistent Todd’s paresis of the right side of the body. A second opinion at another epilepsy center was performed. Ictal SPECT demonstrated regional cerebral hyperperfusion which together with semiology suggested a focus within the SMA-region in the left hemisphere. This epileptic focus  was considered to be secondary to the hamartoma.

The patient described clear difference in the experience involving his ecstatic seizures compared to the “laughing seizures”. The ecstatic seizures were connected to an indescribable bliss, but the gelastic seizures contained a vague “funny feeling”. Heightened emotions seemed to trigger his gelastic seizures.

#### 3.1.1 Semiology changes

After surgery in 2005, the seizures changed, both the gelastic and the ecstatic seizures vanished completely, but the severity of the focal motor seizures increased. Focal seizures with right-sided motor symptoms evolved to bilateral convulsive seizures and became longer and more intense. They remained relatively constant until about 2015 when the patient experienced return of infrequent gelastic seizures and in 2016 return of ecstatic seizures.

### 3.2 Analysis of morphological MRI scans

**Fig 1:**
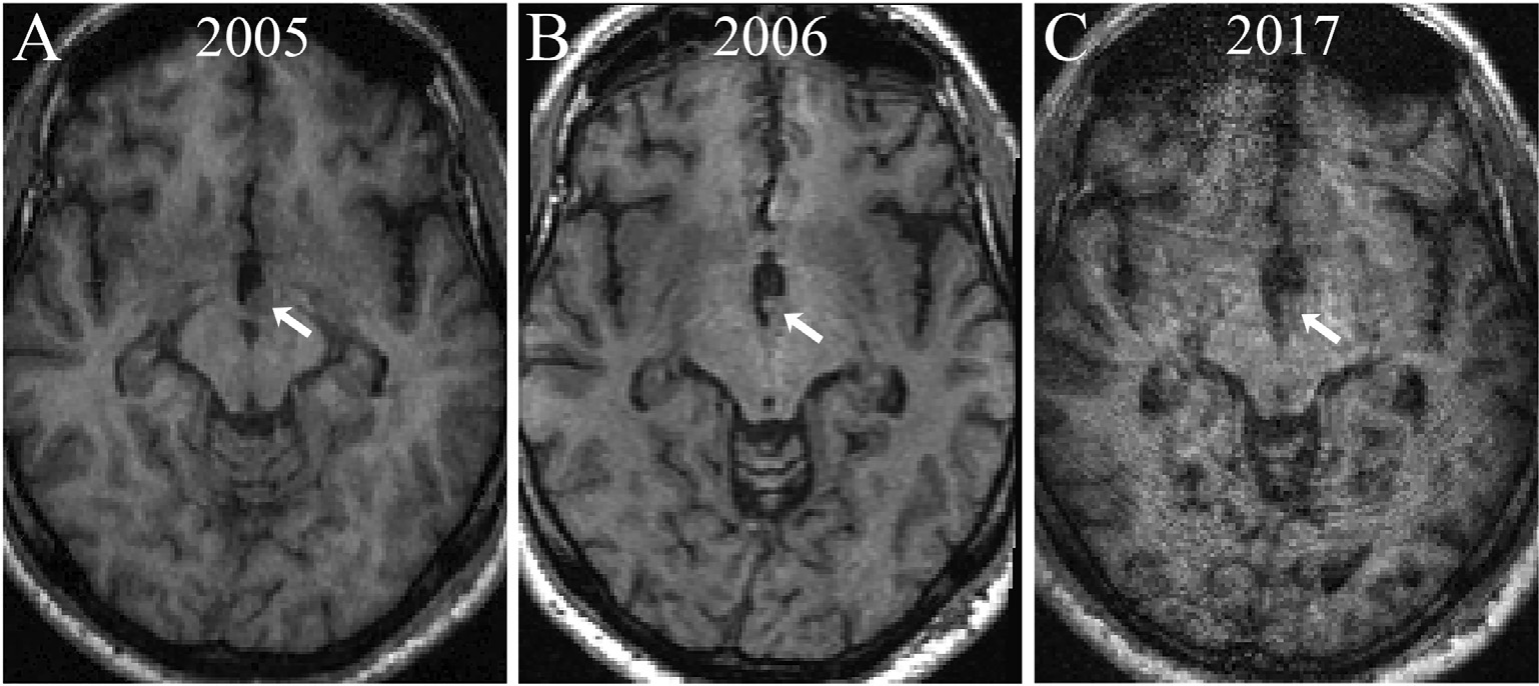
Three anatomic T1-weighted MRI axial images: During presurgical evaluation in 2005 (A) postsurgical evaluation in 2006 (B) and 2017 (C). Note that following initial surgical resection in 2006, the tumor volume was reduced to less than half of the original one. The arrow points to the intraventricular component of the tumor, in relation to the lateral inferior wall of the third ventricle.

According to standard morphological MRI, the tumor was invading the left lateral wall of the third ventricle on its lower border in close proximity to the midline. The tumor volume was 0.50 cm^3^ at the preoperative MRI scan in 2005 with a prominent exophytic component within the third ventricle; the postoperative investigation in 2006 showed 0.20 cm^3^ of tumor remaining within the wall of the third ventricle. Eleven years later the MRI scan showed a stable volume of 0.22 cm^3^. No ischemic lesions or other signal alterations were identified postoperatively (See Fig. 1).

## 4 Discussion

We describe a long-term follow-up report of a patient with a left-sided hypothalamic hamartoma who experienced a dynamic change in gelastic and ecstatic seizures before and after surgery.

### 4.1 Interpretation of semiology

Laughter during childhood as a semiology associated with gelastic seizures, is known to be related to hypothalamic hamartomas [22]. The network involved in the ictal manifestation of laughter has not been fully elucidated. Some authors suggest epileptiform discharges may even involve the cerebellar connections either via the cerebro-ponto-cerebellar pathway or propagation via the thalamus to the brainstem as demonstrated by ictal SPECT [33].

Hypothalamic hamartomas are often associated with multiple seizure types. Gelastic seizures are believed to originate from the hamartoma itself, while seizures of other semiology in such a patient may have onset zones in other cortical areas like the frontal, temporal and insular regions due to secondary propagation or epileptogenesis [34]. The hypothalamus is connected by white matter tracts to several cortex regions including the insula [35], [36]. Subsequently, there is a possibility that in our patient with gelastic seizures, there was an activation of neuronal networks that involve the insula. Considering that gelastic and ecstatic seizures resolved following initial surgical resection, yet focal seizures persisted, one may postulate that the frontal focus now dominated the impaired clinical picture with frequent focal and convulsive seizures. When the gelastic seizures reappeared after many years this appeared to subsequently trigger and realign with redevelopment of ecstatic seizures a year later, reflecting a reactivated neuronal network probably involving the insula.

We also found that the patient’s gelastic seizures were provoked by emotional stimuli. Furthermore, when gelastic seizures occurred, they were often associated with ecstatic seizures. There have been other reports of patients with ecstatic seizures who were able to trigger the seizures by specific memories or emotions [7], [27] and even a minimal stimuli of the hyperexcitable zone would evoke an epileptic discharge in these individuals [37].

## 5 Limitations

Our article has several limitations. Firstly, the patient had several seizure types that changed in frequency over time, so the ecstatic seizures were challenging to classify and quantify. In addition, ictal EEG demonstrated no focal seizure onset zone due to obscuration by muscle artifacts though this is not uncommon in deep midline and brief extratemporal focal seizures. Lastly, radiological follow-ups were limited by VNS though post-operative progression was not identified despite evolution of seizure semiology.

## 6 Conclusion

We describe the longitudinal symptomatology in a patient with gelastic and ecstatic seizures beginning in childhood in relation to a hypothalamic hamartoma. To the best of our knowledge this has not previously been described in detail before. Our case implicates a connection between these seizure types both before and after surgery for a hypothalamic hamartoma. We agree with other reports on ecstatic seizures that ictal semiology is due to propagation of ictal activity between cortical and subcortical regions of the brain and may change over time [6] likely involving the insula and displaying a unique combination of activation/deactivation of cortical areas leading to a changing spectrum of focal seizures [38]. More studies are warranted to better clarify the anatomical-functional networks involved in patients experiencing gelastic or ecstatic seizures.

## 7 Ethics

The study was performed according to the ethical standards of the Helsinki declaration. The study was approved by the ethical committee (ethical permission number 2017/186, Uppsala) and an informed written consent was obtained. The index patient clearly stated his approval of our publication of the clinical data in this article, in written form.

## Conflict of interest

The authors declare that the research was conducted in the absence of any commercial or financial relationships that could be construed as a potential conflict of interest.

## Funding

This research did not receive any specific grant from funding agencies in the public, commercial, or not-for-profit sectors.
